# Polymorphic marker regions support divergence of *Mansonella* sp. “DEUX” and *M. perstans*

**DOI:** 10.1186/s13071-026-07416-y

**Published:** 2026-04-27

**Authors:** Mara Fischer, Miriam Rodi, Prithivi Jung Thapa, Capucine Marie Sicard, Juliana Inoue, Lilith Berner, Pierre Blaise Matsiegui, Carsten Köhler, Andrea Kreidenweiss, Michael Ramharter, Selidji Todagbe Agnandji, Stephan Ossowski, Jana Held

**Affiliations:** 1 Institute of Tropical Medicine Tübingen, University Hospital Tübingen, Tübingen, Germany; 2 Institute of Medical Genetics and Applied Genomics, University of Tübingen, Tübingen, Germany; 3 Centre de Recherches Médicales de la Ngounié, Fougamou, Gabon; 4 Centre de Recherches Médicales de Lambaréné (CERMEL), Lambaréné, Gabon; 5 German Center for Infection Research (DZIF), Partner Site Tübingen, Tübingen, Germany; 6 Center for Tropical Medicine, Bernhard Nocht Institute for Tropical Medicine & I. Department of Medicine, University Medical Center Hamburg-Eppendorf, Hamburg, Germany; 7 German Center for Infection Research Deutsches Zentrum Für Infektionsforschung (DZIF), Partner Sites Hamburg–Lübeck–Borstel–Riems, Hamburg, Germany; 8 Institute for Medical Microbiology, University of Münster, Münster, Germany; 9 Institute for Bioinformatics and Medical Informatics, University of Tübingen, Tübingen, Germany

**Keywords:** *Mansonella* sp. “DEUX”, *Mansonella perstans*, Genotyping, ITS1, *cox1*, *12S* rDNA, *28S* rDNA

## Abstract

**Background:**

Though being prevalent worldwide, *Mansonella* parasites are among the most neglected filarial nematodes. The true prevalence and genetic diversity of this genus have yet to be fully understood. *Mansonella* sp. “DEUX” is a recently described filarial nematode infecting humans and other primates in Gabon and Cameroon, although its status as distinct species has been controversial. We investigated four different polymorphic regions to further explore the genetic differences between *Mansonella* species and to support their status as distinct species.

**Methods:**

We screened whole blood samples collected in EDTA tubes from individuals from rural areas in Gabon for mono-infections with only one *Mansonella* species, either *Mansonella* sp. “DEUX” or *Mansonella perstans*, as determined by quantitative polymerase chain reaction (qPCR) targeting the ITS1 region. We also included nine blood samples from Togo that had been collected as dried blood spots on 903™ protein saver cards and identified as *M. perstans* mono-infection. We further amplified, sequenced, and analyzed three molecular marker regions *cox1*, *12S* rDNA, and *28S* rDNA for their potential to discriminate between the two *Mansonella* species.

**Results:**

In total, 93 mono-infected blood samples were identified. Distinct single-nucleotide polymorphism (SNP) patterns for the two investigated *Mansonella* species were consistently detected in all four loci. The observed nucleotide divergences were comparable to other Onchocercidae family members. Species identification based on the ITS1 marker region was fully concordant with the SNP patterns in all samples. A complete genetic dimorphism could be observed in each of the four marker regions investigated.

**Conclusions:**

The four polymorphic markers, ITS1, *cox1*, *12S* rDNA, and *28S* rDNA, consistently demonstrated clear dimorphism between the two *Mansonella* species. Our results support the classification of *Mansonella* sp. “DEUX” as a distinct, nonrecombining *Mansonella* species within the Onchocercidae family.

**Graphical Abstract:**

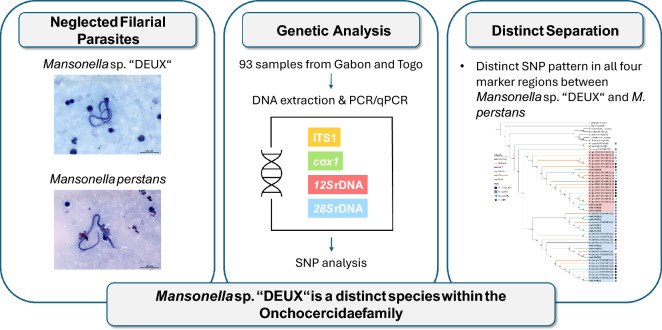

**Supplementary Information:**

The online version contains supplementary material available at 10.1186/s13071-026-07416-y.

## Background

*Mansonella* parasites are filarial nematodes of the Onchocercidae family with possibly the highest worldwide prevalence among all human filarial pathogens [1]. Nevertheless, infections with *Mansonella* species are among the most neglected tropical diseases. Until recently, three species have been known to infect humans. While *M. perstans* is widespread in sub-Saharan Africa and South and Central America, *M. streptocerca* seems to be limited to the African continent and *M. ozzardi* to Latin America [1]. *Mansonella perstans* and *M. ozzardi* are described as blood-dwelling species, while *M. streptocerca* is a skin-dwelling species.

In 2015, a potential new blood-dwelling *Mansonella* species called *Mansonella* sp. “DEUX” was described. It was later found to be the most prevalent filarial species in rural Gabon, infecting 35% of individuals participating in a cross-sectional study [2]. *Mansonella* sp. “DEUX” differed from *M. perstans* in the ITS1 region, the region targeted by polymerase chain reaction (PCR) diagnostics [3], and was confirmed as a distinct *Mansonella* species in 2023 on the basis of whole-genome analysis [4]. Moreover, this was supported by Gaillard et al., who investigated stool samples of nonhuman primates in Gabon and Cameroon for presence of *Mansonella* DNA on the basis of the two genes *12S* rDNA and *cox1* [5]. They found *Mansonella* sequences that clustered separately and were later confirmed to be *Mansonella* sp. “DEUX” [4]. This indicates that similar to *M. perstans*, *Mansonella* sp. “DEUX” infects both humans and nonhuman primates and is prevalent in Gabon and Cameroon. No additional endemic regions have been identified for *Mansonella* sp. “DEUX” so far [6].

*Mansonella* species belong to the Onchocercidae family, as do *Loa loa* or *Onchocerca volvulus*, with which they are often co-endemic. Historically, the description of species and their systematics was based on microscopic morphological descriptions. With the advent of molecular methods, phylogenetic relationships within the Onchocercidae family are increasingly being reconstructed using molecular data rather than morphological descriptions. In 2015, a multilocus sequencing approach based on seven nuclear and mitochondrial loci proposed a division of this family into distinct clades, placing *Mansonella* species in close relationship with *Loa*, *Brugia*, and *Wuchereria* [7], with which they are often co-endemic. Phylogenetic studies on nematodes, including *Mansonella* species, are based on the two mitochondrial genes *cox1* and *12S* rDNA [5, 8–11]. The *cox1* gene has been particularly effective for species differentiation across most metazoan species [12–14]. The nuclear *28S* rDNA gene is another potential marker region included in multilocus sequencing approaches investigating the Onchocercidae phylogeny [7, 15].

We recently showed that *M. perstans* and *Mansonella* sp. “DEUX” are two sympatric species on the basis of the whole-genome sequences of three samples. To further validate this finding, we investigate in this work whether sequence dimorphisms are consistently observed between the species in a larger number of samples and at different genetic loci and evaluate the occurrence of potential interbreeding between the proposed species. We analyzed four different genetic regions in 93 field samples from endemic regions in Gabon and Togo. In addition to the ITS1 region, which is used to differentiate between both species in quantitative PCR analysis, we analyzed the two mitochondrial genes *cox1* and *12S* rDNA and the nuclear region *28S* rDNA. Moreover, we included previously published sequences from the database for comparative phylogenetic analysis.

## Methods

### Study sampling and DNA extraction

In total, 93 samples identified with only one *Mansonella* species, either *Mansonella* sp. “DEUX” or *M. perstans*, hereby referred to as mono-infected sample, from four different study cohorts in Gabon (84 samples) and Togo (9 samples), were included in this study. A detailed explanation of the sample collection for each study is described in Additional File 2: Supplementary Table S1. The diagnosis of *Mansonella* mono-infection was based on molecular diagnostics using ITS1 qPCR. All studies were approved by the Institutional Ethics Committee of CERMEL or by the Togolese Bioethics Committee for Research in Health. Written informed consent was obtained from all adult participants or their legal representatives. Further details on the study populations are provided in Additional File 2: Supplementary Table S1.

### Amplification of four genetic marker regions: ITS1, *cox1*, *12S* rDNA, and *28S* rDNA

Markers that have previously been used for multilocus sequence typing of onchocercid species [7] were selected on the basis of the largest availability of sequence data from other onchocercid species and especially filarial species in the database, allowing for more robust comparisons. The ITS1 locus was preamplified in a conventional PCR assay with a total volume of 25 µl, followed by a qPCR using the LightCycler^®^ 480 II from Roche. Two specific probes differentiated between *M. perstans* and *Mansonella* sp. “DEUX”, while the primers targeted a conserved region of the ITS1 locus, as previously published [2]. SensiFAST™ Probe NoROX Kit (Meridian Bioscience) was used in a total reaction volume of 10 µl. Cycling conditions and PCR reaction mix conditions for the preamplification and the qPCR are listed in Additional File 2: Supplementary Table S2. Cq values and amplification curves were analyzed using the LightCycler^®^ 480 Software.

*Cox1*, *12S* rDNA, and *28S* rDNA loci were amplified in a nested PCR approach. All reactions were run in a total volume of 15 µl. The respective cycling and reaction mix conditions are listed in Additional File 2: Supplementary Table S2. The following controls were used in all PCR runs: nontemplate control (H_2_O), negative control (DNA from noninfected human whole blood), positive control (DNA from *O. volvulus*, *L. loa*, *M. perstans*, or *Mansonella* sp. “DEUX”). Amplicons were analyzed using automated gel capillary electrophoresis (QIAxcel, QIAGEN) using the AM420 method. A threshold of 5% was used to distinguish true- from false-positive peaks in the electropherogram. Specific QIAxcel settings for each gene are listed in Additional File 2: Supplementary Table S3.

### Sanger sequencing

Amplicons that were subjected to Sanger sequencing were purified using exoSAP-IT (Thermo Fischer Scientific) or GFX TM PCR DNA and Gel Band Purification Kit (GE Healthcare). The respective forward and reverse primers from the nested PCR were added at 5 µM, and the sample was sent to Eurofins Genomics for further sequencing.

### Phylogenetic analysis

Sequences were analyzed using Geneious Prime software (version 2025.1.2). Raw sequences were trimmed and aligned to the respective reference sequences (Additional File 2: Supplementary Table S4). No reference was available for *28S* rDNA from *Mansonella* sp. “DEUX”. A quality threshold of % high quality (HQ) > 65% was set for all sequences. Consensus sequences were used for further analysis. Single forward or reverse sequences were used alone if the respective complementary sequence did not meet the %HQ threshold of > 65%. To ensure the reliability of the detected SNPs in the sequences, we applied three stringent criteria that all had to be fulfilled: The SNP had to be identified in more than one sample; it had to be found in both the forward and reverse reads; and it had to be called with a Phred score of at least 50 in both forward and reverse reads, giving a joint probability of 10^−10^ for incorrect base calling. Additional sequences obtained from the National Center for Biotechnology Information (NCBI) nucleotide database were used alongside the generated sequences to achieve higher confidence clade separation and check reproducibility across studies.

Sequences were aligned using MAFFT aligner [16]. The subsequent alignment files were trimmed using TrimAl to remove any sequences that did not have any homologous sites in the alignment [17]. ModelFinder was used to find the optimal reconstitution model for each alignment, on the basis of the Bayesian information criterion (BIC) [18]. All phylogenetic trees were generated using 1000 ultrafast bootstrap (UFBoot), and trees were rooted using *Filaria latala* as an outgroup [19]. The following models of substitution were chosen: *12S* rDNA: HKY + F + G4; *28S* rDNA: K3Pu + F + G4; and *cox1*: TIM3 + F + I*G4. Trees were generated using IQ-Tree [20]. The full codes for the tree construction can be found in GitHub (https://github.com/jungey-prithivi/treestuff).

Percent identity matrices were generated from alignments of *cox1*, *12S* rDNA, and *28S* rDNA for each of their sequence sets, using pairwise comparisons obtained from the MAFFT multiple sequence alignment for *M. perstans* and *Mansonella* sp. “DEUX”. Interspecific differences were calculated on the basis of the following formula:

$$100 - \left( {\frac{{{\mathrm{Number}}\; {\mathrm{of}}\;{\text{ identical}} \;{\mathrm{positions}}}}{{{\mathrm{alignment}} \;{\mathrm{length}}}} \times 100} \right)$$

All sequences generated within this work are published in GenBank with the following accession nos.: *cox1* (PX533650–PX533662), *28S* rDNA (PX619923–PX619924), *12S* rDNA (PX619925–PX619929).

## Results

### ITS1 locus as primary marker of differentiation

We identified 31/93 individuals with a mono-infection of *M. perstans* and 62/93 individuals with *Mansonella* sp. “DEUX” on the basis of ITS1 qPCR. *Mansonella* sp. “DEUX” was exclusively detected in samples from Gabon. Amplification success and Cq values were comparable between species (Additional File 2: Supplementary Tables S5 and S6).

Next, we analyzed three additional marker regions for their dimorphism between the two species: *cox1*, *28S* rDNA, and *12S* rDNA. All 93 samples were amplified and sequenced. Both amplification and sequencing rate were reduced for the *12S* rDNA gene compared with *cox1* and *28S* rDNA. Further details can be found in Additional File 2: Supplementary Table S6.

In total, we analyzed 16, 22, and 12 sequences from 31 *M. perstans* samples for *cox1*, *28S* rDNA, and *12S* rDNA, respectively. For the 62 *Mansonella* sp. “DEUX” samples, we analyzed 46, 45, and 13 sequences for the three marker regions.

### Marker regions *cox1*, *28S* rDNA, and *12S *rDNA confirm *Mansonella* species discrimination on the basis of the ITS1 locus

The dimorphisms we found in the sequences of the three markers *cox1*, *28S* rDNA, and *12S* rDNA all validate the classification into either *M. perstans* or *Mansonella* sp. “DEUX” based on the ITS1 marker. We saw a consistent distinction between the two species with SNPs in three marker regions reliably distinguishing *M. perstans* from *Mansonella* sp. “DEUX”, aligned with ITS1-based identification (Table 1). However, the degree of divergence and SNP patterns differed among the three loci. The positions of the respective SNPs are listed in Additional File 2: Supplementary Table S7.

**Table 1:** Overview of single-nucleotide polymorphisms between *M. perstans* and *Mansonella* sp. "DEUX"

Gene	Length of the analyzed sequences	Number of SNPs between *M. perstans* and *Mansonella* sp. “DEUX”	Percentage (%) of SNPs between *M. perstans* and *Mansonella* sp. “DEUX”	Interspecific differences in %	Previously observed nucleotide divergences between onchocercid species in %
*cox1*	619 bp	35	5.6	7.9–9.9	4.5–13 [9, 21–23]
*28S* rDNA	519 bp	3	0.6	0.4–0.8	NA
*12S* rDNA	394 bp	13	3.3	3.4–4.2	> 2 [23]

The length of the analyzed sequence refers to the respective reference sequence. bp, base pairs

The *cox1* region showed the highest level of genetic diversity with the most distinct SNP pattern between both species. Along the 619-bp *cox1* amplicon, 35 SNPs were identified between the two species and found in all analyzed samples (62/62). The *28S* rDNA region was highly conserved with three SNPs that were consistently found (67/67 samples) along the 519-bp amplicon differentiating between *M. perstans* and *Mansonella* sp. “DEUX”. In addition, 13 SNPs consistent across all sequences (25/25 samples) were identified within the 394-bp amplicon in *12S* rDNA. The level of nucleotide divergence between the two species is comparable to or even superior to that observed in other onchocercid species (Table 1). Sequence alignments for all three loci can be found in Additional File 1. Percent identity matrices and interspecific differences are presented in Additional File 2: Supplementary Tables S8–S10.

### Phylogenetic trees based on *cox1*, *12S* rDNA, and *28S *rDNA separate *M. perstans* and *Mansonella* sp. “DEUX” into distinct clusters

We included 80 additional *cox1* sequences (48 *Mansonella* sp. “DEUX”; 32 *M. perstans)*, 49 additional *12S* rDNA sequences (28 *Mansonella* sp. “DEUX”; 21 *M. perstans)* from both *Mansonella* species, as well as 1 additional available *M. perstans* sequence for *28S* rDNA retrieved from GenBank in the construction of phylogenetic trees. Accession numbers of all the included sequences are listed in Additional File 2: Supplementary Table S11. All SNPs described above were confirmed in additional sequences in all marker regions. Additional sequences that were derived from the NCBI sequences were added to the phylogenetic tree. Additional File 2: Supplementary Table S12 provides an overview of the reconstitution of the respective genotypes, including the accession numbers.

**Fig. 1:**
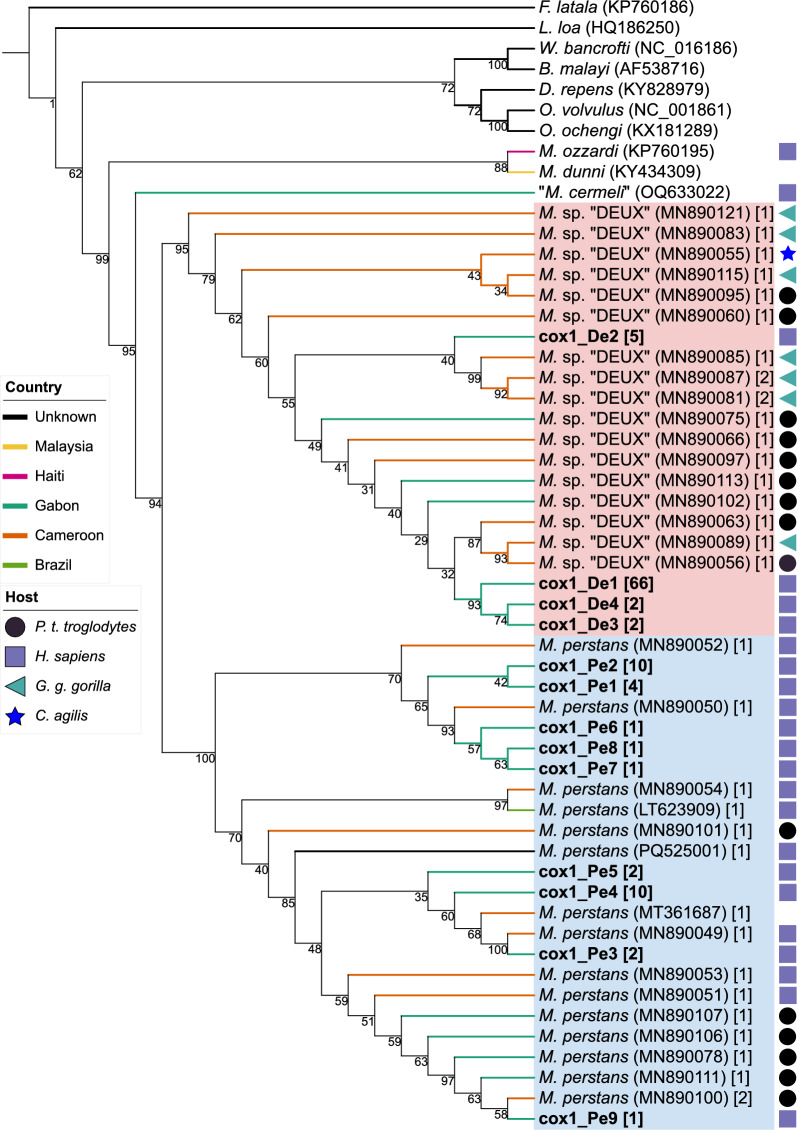
Phylogenetic tree based on *cox1*, including sequences from *M. perstans* (blue), *Mansonella* sp. “DEUX” (red), and ten additional nematode species. Bold letters indicate sequences that were generated within this study. Accession numbers are displayed in round parenthesis. The number in square parenthesis indicates the number of sequences that support the respective genotype. Colored branches indicate the country of origin, and symbols indicate the host

**Fig. 2:**
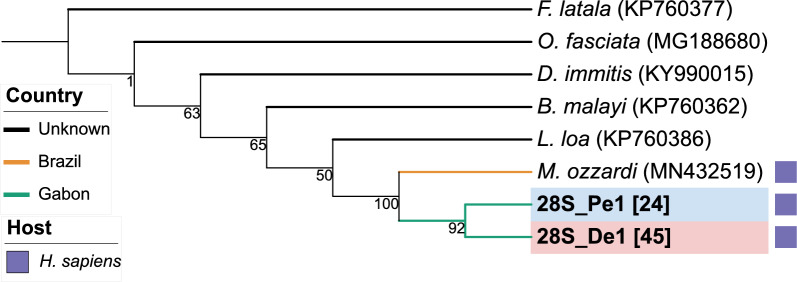
Phylogenetic tree based on *28S* rDNA. *M. perstans* sequences are highlighted in blue and *Mansonella* sp. “DEUX” sequences in red. Bold letters indicate sequences that were generated within this study. Accession numbers are displayed in round parenthesis. The number in square parenthesis indicates the number of sequences that support the respective genotype. Colored branches indicate the country of origin, and symbols indicate the host

**Fig. 3:**
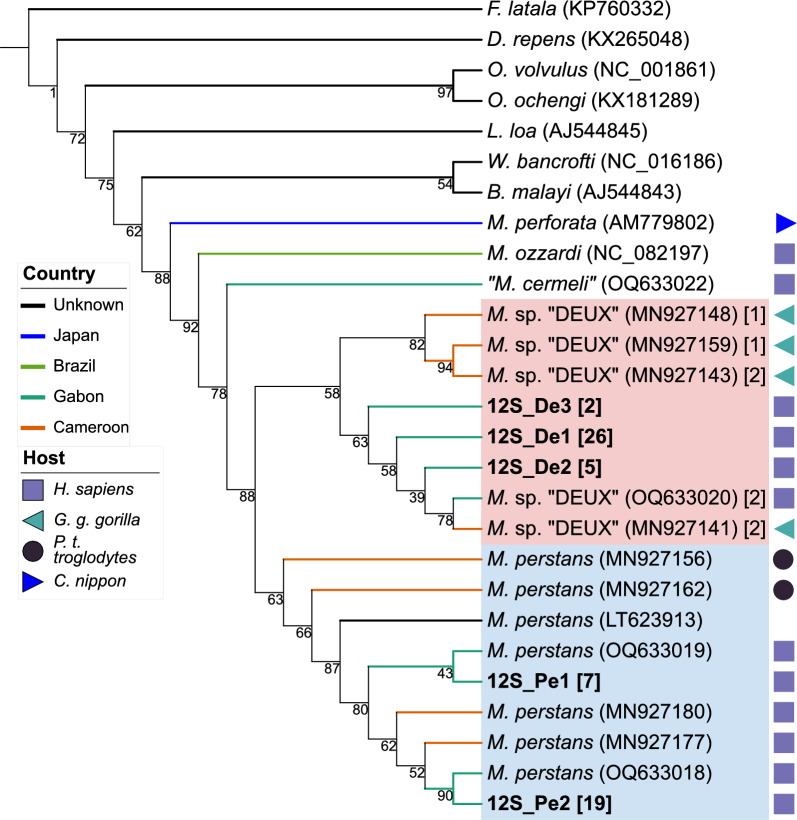
Phylogenetic tree based on *12S* rDNA. *M. perstans* sequences are highlighted in blue and *Mansonella* sp. “DEUX” sequences in red. Bold letters indicate sequences that were generated within this study. Accession numbers are displayed in round parenthesis. The number in square parenthesis indicates the number of sequences that support the respective genotype. Colored branches indicate the country of origin, and symbols indicate the host

To visualize the filarial systematics, focusing on *M. perstans* and *Mansonella* sp. “DEUX”, we constructed phylogenetic trees on the basis of the three loci: *cox1* (Fig. 1), *28S* rDNA (Fig. 2), and *12S* rDNA (Fig. 3). Additional sequences from other filarial species were included as indicated in the respective phylogenies.

**Fig. 4:**
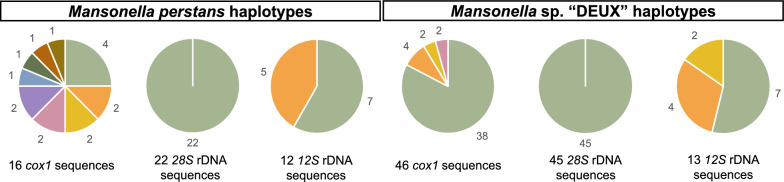
Distribution of *Mansonella* species haplotypes. Different colors represent different haplotypes. The number of sequences for each haplotype is indicated

The phylogenetic tree based on *cox1* shows a distinct *Mansonella* clade with two separate clades: one for *Mansonella* sp. “DEUX” (*N* = 21 genotypes) and one for *M. perstans* (*N* = 24 genotypes), well-supported by a bootstrap value of 94%. The two *M. perstans* genotypes, cox1_Pe1 and cox1_Pe2, composed of sequences from Togo exclusively, cluster together within the *M. perstans* branch. Bootstrap support for the separation between *M. ozzardi* and *M. dunni* to *M. perstans* and *Mansonella* sp. “DEUX” is 95%. In the *28S* rDNA tree, the two identified genotypes (*M. perstans*: *N* = 1, *Mansonella* sp. “DEUX”: *N* = 1) form distinct clades with high bootstrap support. The *12S* rDNA tree shows a distinct *Mansonella* clade with two separate clades for *M. perstans* (*N* = 9) and *Mansonella* sp. “DEUX” (*N* = 8) with lower bootstrap support than in the *cox1* region (36%). A separate *Mansonella* clade, including other *Mansonella* species (*M. perforata*, *M. ozzardi*, and "*M. cermeli"*) branches with high bootstrap support (94%).

To conclude, *M. perstans* and *Mansonella* sp. “DEUX” form separate clusters in all three loci both to each other as well as to *M. ozzardi*. Our observations not only support the status of *Mansonella* sp. “DEUX” as a distinct species but also validate the use of the ITS1 region for species differentiation.

### Distinct haplotypes occurred within the two *Mansonella* species in the different marker regions

We observed different degrees of nucleotide diversity within the two species in the three marker regions (Table 2). Overall, diversity was higher within *M. perstans* than *Mansonella* sp. “DEUX”.

**Table 2:** Overview of the observed number of sequences and haplotypes for each of the two *Mansonella* species found among the three marker regions *cox1*, *28S* rDNA, and *12S* rDNA

Marker region	*M. perstans*		*Mansonella* sp. “DEUX”	
	Sequences	Haplotypes	Sequences	Haplotypes
*cox1*	16	9	46	4
*28S* rDNA	22	1	45	1
*12S* rDNA	12	2	13	3

With nine *cox1* haplotypes in 16 *M. perstans* sequences and four *cox1* haplotypes in 46 *Mansonella* sp. “DEUX” sequences, we could describe seven and two new haplotypes, respectively, while two haplotypes of each species had already been reported previously in GenBank.

Using the *12S* rDNA marker, we were able to detect one new haplotype in each of the two species. For the marker regions *cox1* and *12S* rDNA, one major haplotype and multiple minor haplotypes were detected. We found only one haplotype each within the *28S* rDNA sequences for both species. Figure 4 displays the different haplotypes found within the sequences of each species.

We compared the nucleotide divergence in percentages between each haplotype from all three markers with the respective reference sequence within the two *Mansonella* species (Additional File 2: Supplementary Tables S13 and S14). We observed that the mean nucleotide divergence among all *Mansonella* sp. “DEUX” haplotypes from *12S* rDNA and *cox1* was lower than for *M. perstans*.

### Haplotypes can be associated with geographical origin

Both *M. perstans* and *Mansonella* sp. “DEUX” have been observed to infect nonhuman primates in Gabon and Cameroon [4, 5]. To investigate a potential association of different haplotypes with ecological factors such as host species or country of origin, haplotypes from samples with different ecological factors were compared. Our study samples were of human origin exclusively. The database from GenBank comprised 85 additional *Mansonella* spp. sequences from nonhuman primates, which were added to our phylogenetic analysis (32 sequences for *12S* rDNA and 53 for *cox1)*. Overall, 74 sequences from Cameroon were added from GenBank (29 sequences for *12S* rDNA and 45 sequences for *cox1*) to our 84 samples from Gabon and 9 samples from Togo. We observed no association between host origin and haplotypes. Major haplotypes were shared between samples of different host origins (human or nonhuman primates) for all three loci. We observed distinct *cox1* and *12S* rDNA haplotypes in *M. perstans* samples depending on the origin. All seven analyzed *M. perstans* samples from Togo shared a distinct new *12S* rDNA haplotype (12S_Mpe1). In *cox1*, the samples from Togo shared two distinct haplotypes comprised of four and two sequences, respectively (cox1_Mpe1 and cox1_Mpe2), which differed at 14 nucleotide positions (2.8%). The haplotype cox1_Mpe1 has not been observed before, whereas cox1_Mpe2 was found in eight additional sequences from GenBank, seven of which originated from West Africa. Additional *cox1* sequences from GenBank originated from Central African countries and mapped with haplotypes from our study samples from Gabon.

## Discussion

It has long been known that different *Mansonella* species infect humans and nonhuman primates in sub-Saharan Africa and South America. Historically, species identification has relied primarily on morphological characteristics of microfilaria and adult worms [24, 25]. Advances in molecular methodologies such as multilocus genotyping have facilitated species delineation by revealing genetic variation across multiple genomic regions. Recently, we described *Mansonella* sp. “DEUX” as a distinct species based on whole-genome data, infecting humans in rural Gabon with a prevalence of 35% [2–4]. In this study, we expanded upon previous findings by comparing a larger set of samples of the two sympatric species—*Mansonella* sp. “DEUX” and *M. perstans*—across four polymorphic loci: ITS1, *Cox1*, *12S* rDNA, and *28S* rDNA.

Using 93 mono-infected samples, we assessed species identification on the basis of these markers. Amplification and sequencing success varied by targeted genomic region, consistent with previous reports that also reported difficulties sequencing the *12S* rDNA region in *Mansonella* [5, 26]. Owing to limited reference sequences, primer–template mismatches cannot be excluded, although the primers target a conserved filarial region, and *12S* rDNA is widely applied in phylogenetic studies in metazoan species in general and in Onchocercidae [7, 11, 27, 30].

We observed clear and consistent SNP patterns distinguishing the two species across all three loci, which supports the validity of ITS1 as a diagnostic marker. The concordant separation across mitochondrial (*Cox1* and *12S* rDNA) and nuclear (*28S* rDNA) loci reduces the likelihood of locus-specific bias. Phylogenetic analyses demonstrated robust clustering of the two species without evidence for recombination. The level of nucleotide divergence between the two species was comparable to differences observed among other onchocercid species. As expected, the divergence was greater in mitochondrial genes (*cox1* and *12S* rDNA) than in the nuclear gene *28S* rDNA [27]. The phylogenetic analysis revealed distinct clustering of both species in all three regions. Our analysis was consistent with the multilocus phylogeny proposed by Lefoulon et al., who placed *Mansonella* within clade ONC5, alongside *Loa* and *Brugia*. Our results showed clear and distinct clustering of the *Mansonella* species, on which we focused, as well as overall similar clustering of the outgroups compared with Lefoulon et al. Although our analysis included only three of the seven loci used in that study, the results support the value of combining mitochondrial and nuclear markers for robust phylogenetic interference.

Divergence among *M. perstans* isolates from Gabon, Cameroon, and Togo was lower than between *M. perstans* and *Mansonella* sp. “DEUX” in Gabon. Distinct *M. perstans* haplotypes detected in samples from Togo suggest geographically structured transmission zones with limited interbreeding. In human and nonhuman primate (NHP) samples from Gabon and Cameroon, no host-specific genotypes were detected, indicating an absence of strict host specificity. *Mansonella* sp. “DEUX” was exclusively detected in human samples from Gabon and NHPs from Cameroon and Gabon. Until now, there are no other reports of *Mansonella* sp. “DEUX” outside this area [6]. Our analysis of sequences previously collected by Gaillard et al. further supports the designation of the originally unspecified sequences as *Mansonella* sp. “DEUX”, as previously proposed [4, 5], thereby making it the only *Mansonella* species alongside *M. perstans* that could be detected in the blood and stool of NHPs. Whether it corresponds to the ape-associated *M. rodhaini* remains uncertain owing to the absence of molecular data for that species [24].

Other *Mansonella* species also show zoonotic potential. *M. ozzardi*, common in the Americas, has recently been detected in racoons and coatis [26, 28]. Other *Mansonella* species infect NHPs as well as other mammals [29]. Given the sympatry of humans and animals in endemic regions, zoonotic transmission may pose an underrecognized public health risk [28]. Further research is necessary to evaluate the full scope of its zoonotic potential and effects.

The consistent genetic distinctions observed across four genetic regions confirm *Mansonella* sp. “DEUX” as a distinct species from *M. perstans*. Further evidence of reproductive isolation according to the Biological Species Concept would strengthen this classification. As both species often occur in the same host, a biological species barrier prohibiting crossing is very likely, though unproven, owing to the lack of a cell culture system for adult parasites, which would enable cross-mating experiments [30].

Further research is necessary to investigate potential vector differences, host- or life-cycle variation, and *Wolbachia* endosymbiont diversity to better understand their evolutionary separation and guide targeted treatment strategies for mansonellosis [6].

## Conclusions

Consistent genetic polymorphisms in ITS1 and three additional marker regions confirm *Mansonella* sp. “DEUX” as a distinct species from *M. perstans* and validate ITS1 as a reliable diagnostic marker. Our findings strengthen the molecular framework for understanding the phylogeny, epidemiology, and potential health burden of *Mansonella* species. Despite being the most prevalent filarial infection, *Mansonella* parasites remain highly neglected, and the recent discovery of *Mansonella* sp. “DEUX” highlights the likelihood of underestimating their true impact. Broader screening for this species outside Gabon will help clarify its distribution and burden of disease. Validating molecular markers for distinct species classification is crucial for improving knowledge of *Mansonella* taxonomy, endemicity, and public health significance.

## Supplementary Information

Additional file 1 : Alignments of three marker regions *cox1, 28S *rDNA,*12S *rDNA, obtained from *Mansonella perstans* and *Mansonella* sp. “DEUX” samples, to the respective reference sequences (marked in light yellow).

Additional file 2 : Number of sequences used for the respective genotype in the phylogenetic trees.

## Data Availability

All data are available in GenBank under the following accession nos.: *cox1* (PX533650–PX533662), *28S* rDNA (PX619923–PX619924), *12S* rDNA(PX619925–PX619929).
