# Supplementary material for: Polymorphic marker regions support divergence of Mansonella sp. “DEUX” and M. perstans
Source: Parasit Vectors. 2026 Apr 27;19:183. doi: 10.1186/s13071-026-07416-y (PMC13112848; doi:10.1186/s13071-026-07416-y)
Supplement: Supplementary file 1 — Additional file 1 : Alignments of three marker regions cox1, 28S rDNA,12S rDNA, obtained from Mansonella perstans and Mansonella sp. “DEUX” samples, to the respective reference sequences (marked in light yellow). [file 13071_2026_7416_MOESM1_ESM.docx]

**Additional file 1**: Alignments of three marker regions *cox1, 28S* rDNA, *12S* rDNA, obtained from *Mansonella perstans* and *Mansonella* sp. “DEUX” samples, to the respective reference sequences (marked in light yellow).

***Cox1***


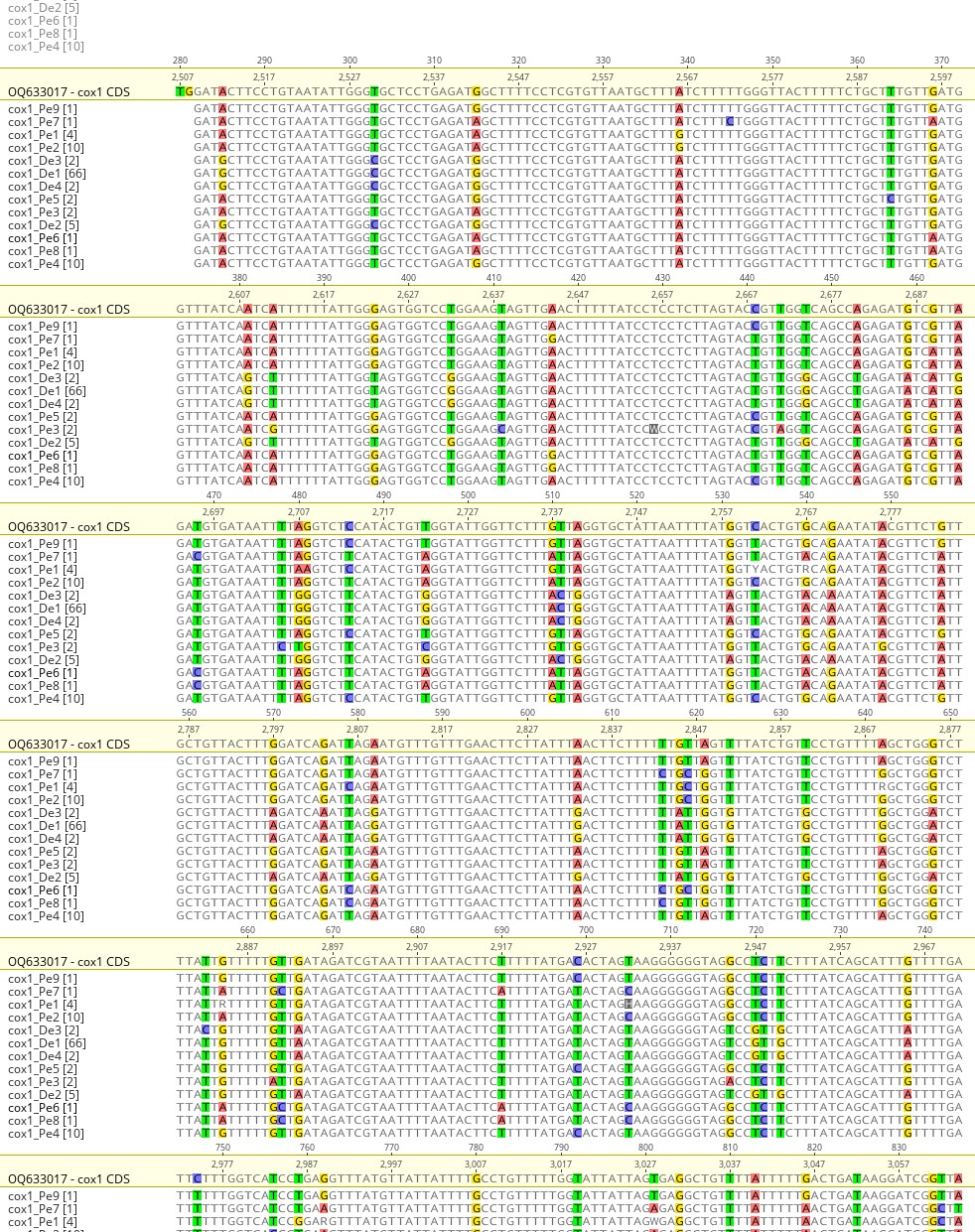


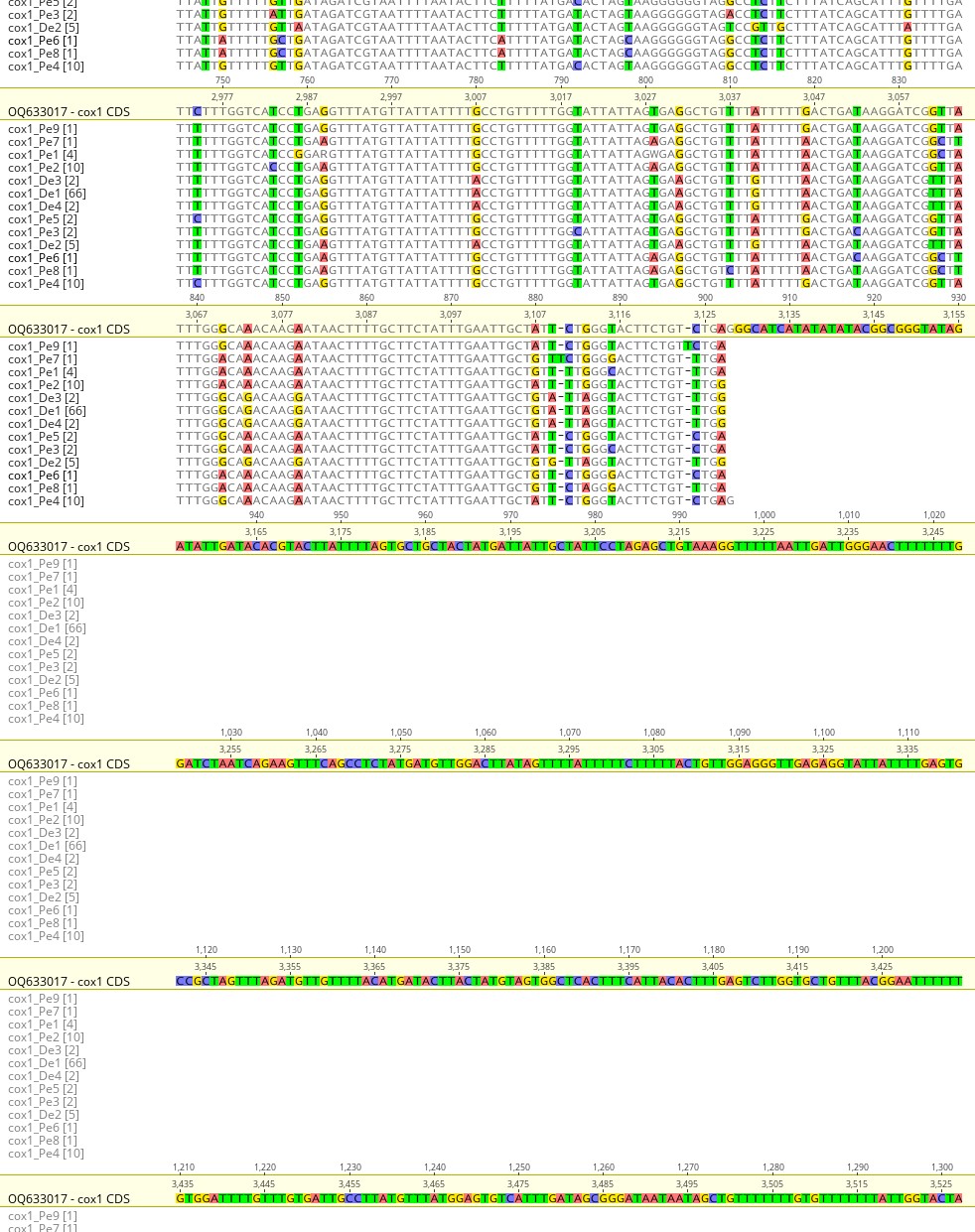


***28S* rDNA**

**
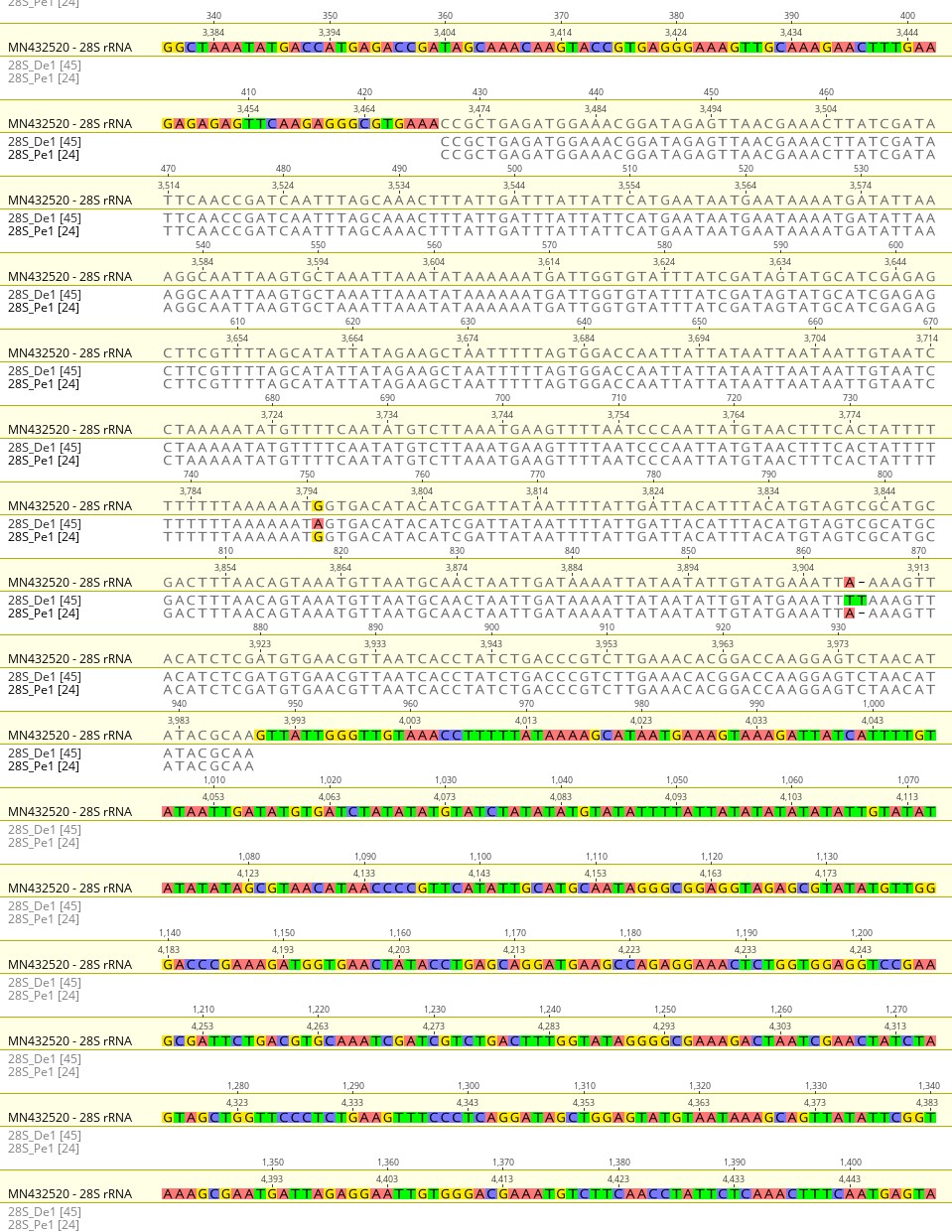
**

***12S* rDNA**

**
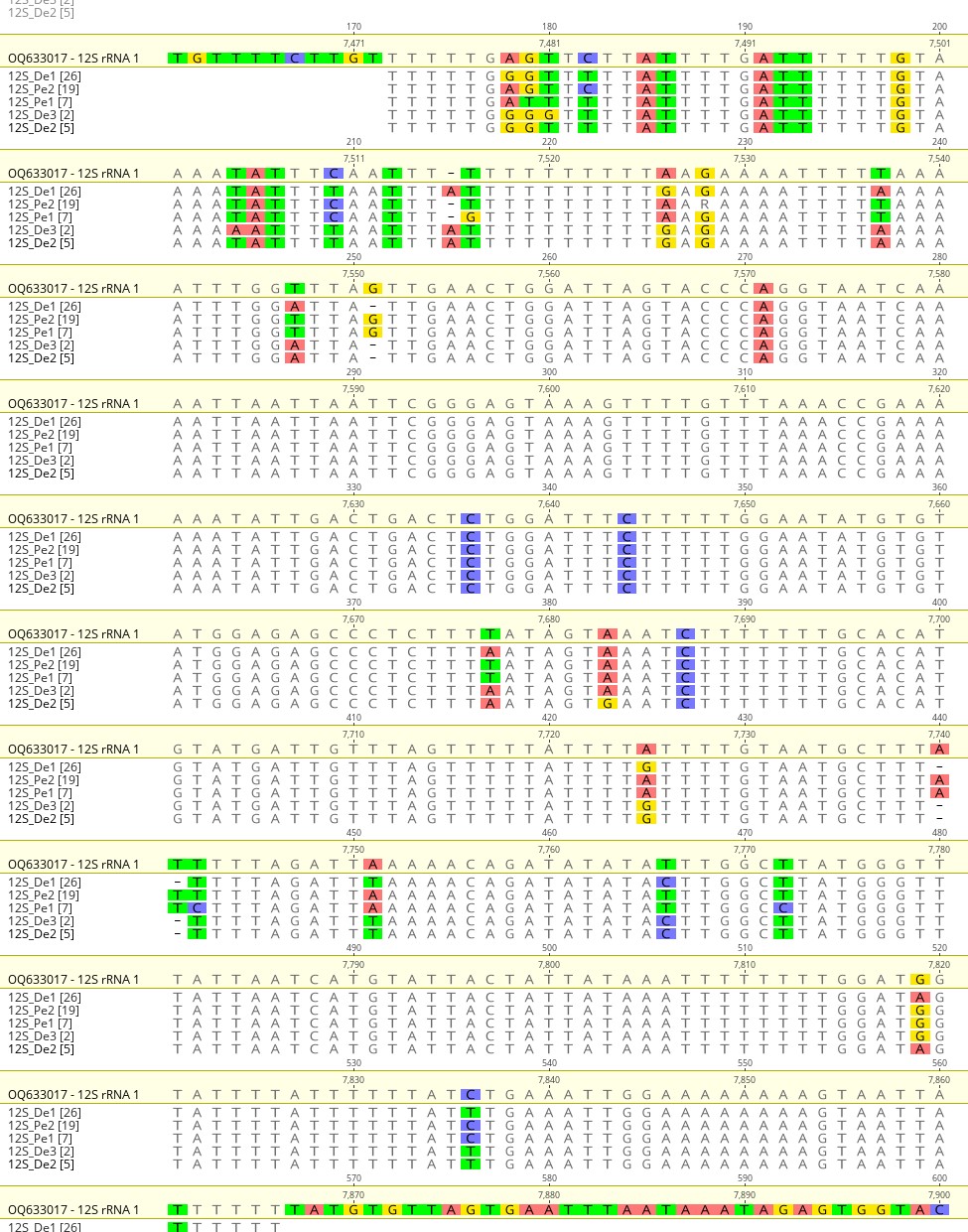
**

**
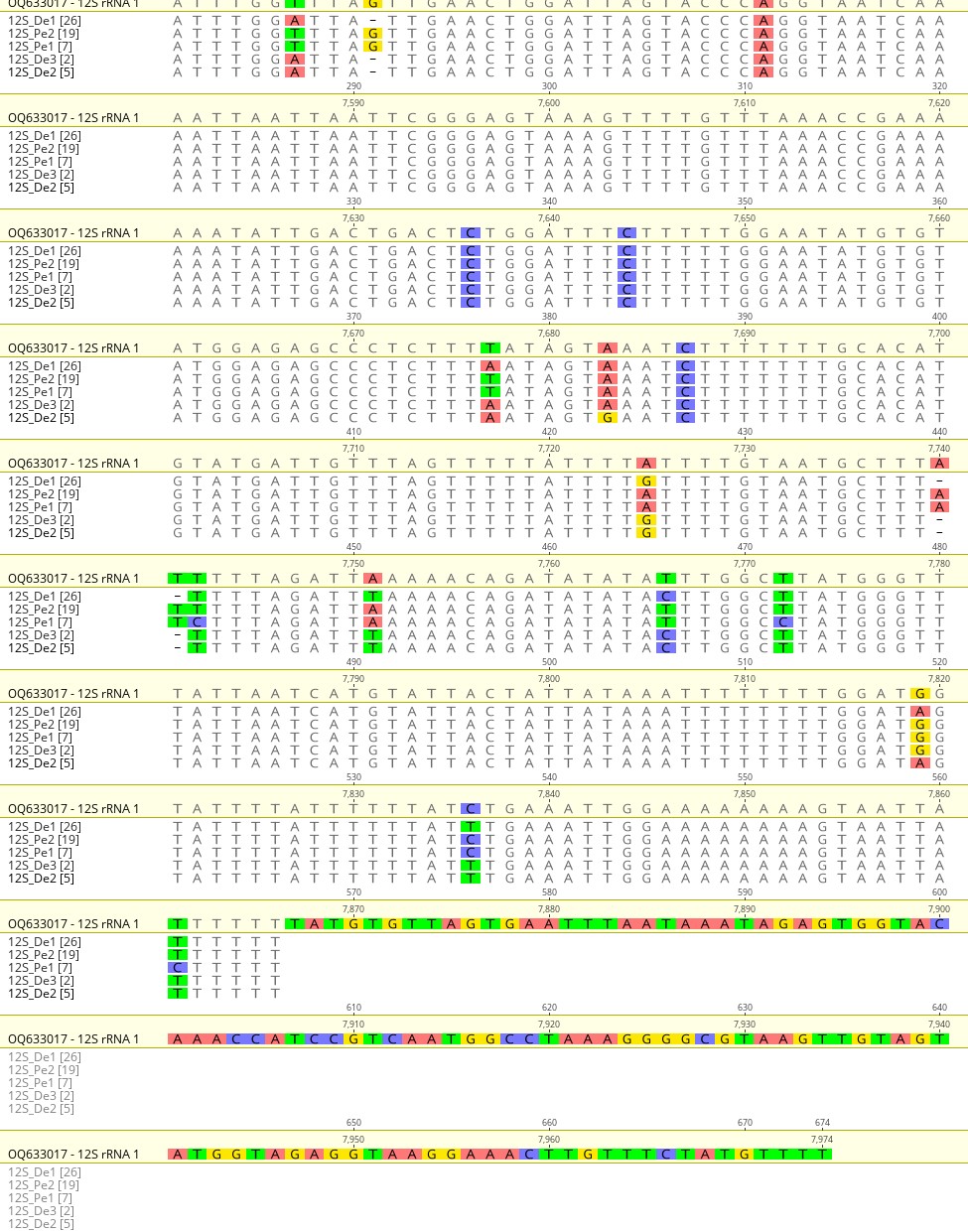
**
